# Combining Mathematical Models With Experimentation to Drive Novel Mechanistic Insights Into Macrophage Function

**DOI:** 10.3389/fimmu.2019.01283

**Published:** 2019-06-06

**Authors:** Joanneke E. Jansen, Eamonn A. Gaffney, Jonathan Wagg, Mark C. Coles

**Affiliations:** ^1^Mathematical Institute, University of Oxford, Oxford, United Kingdom; ^2^Kennedy Institute of Rheumatology, University of Oxford, Oxford, United Kingdom; ^3^F. Hoffmann-La Roche, Basel, Switzerland

**Keywords:** macrophages, monocytes, IBD, mechanistic mathematical models, *in silico* experimentation

## Abstract

This perspective outlines an approach to improve mechanistic understanding of macrophages in inflammation and tissue homeostasis, with a focus on human inflammatory bowel disease (IBD). The approach integrates wet-lab and *in-silico* experimentation, driven by mechanistic mathematical models of relevant biological processes. Although wet-lab experimentation with genetically modified mouse models and primary human cells and tissues have provided important insights, the role of macrophages in human IBD remains poorly understood. Key open questions include: (1) To what degree hyperinflammatory processes (e.g., gain of cytokine production) and immunodeficiency (e.g., loss of bacterial killing) intersect to drive IBD pathophysiology? and (2) What are the roles of macrophage heterogeneity in IBD onset and progression? Mathematical modeling offers a synergistic approach that can be used to address such questions. Mechanistic models are useful for informing wet-lab experimental designs and provide a knowledge constrained framework for quantitative analysis and interpretation of resulting experimental data. The majority of published mathematical models of macrophage function are based either on animal models, or immortalized human cell lines. These experimental models do not recapitulate important features of human gastrointestinal pathophysiology, and, therefore are limited in the extent to which they can fully inform understanding of human IBD. Thus, we envision a future where mechanistic mathematical models are based on features relevant to human disease and parametrized by richer human datasets, including biopsy tissues taken from IBD patients, human organ-on-a-chip systems and other high-throughput clinical data derived from experimental medicine studies and/or clinical trials on IBD patients.

## Introduction

Macrophages are heterogeneous cells with key functions in inflammatory immune responses, tissue homeostasis, and immune regulation. They are a first line of defense against pathogens, and, play a major role in maintaining tissue integrity by accelerating repair processes (1). Macrophages are also involved in the pathogenesis and progression of human inflammatory diseases including rheumatoid arthritis (RA), atherosclerosis, and inflammatory bowel disease (IBD). Common polymorphisms that confer disease susceptibility and Mendelian genetic disorders that can present with IBD and RA clearly suggest an important role for macrophage signaling pathways. Loss of function defects in IL-10 signaling induce early onset IBD with complete penetrance and in mouse models macrophage specific loss of IL10R expression causes the spontaneous development of severe colitis (2, 3). Monocyte-derived macrophages are also major sources of inflammatory cytokines such as TNF-α, IL-12/23, and IL-6, all therapeutic targets in IBD and/or RA (4).

Despite genetic and pharmacological evidence that macrophages are important in IBD pathophysiology, the mechanistic details of this role remain to be fully elucidated. For example, the complex intracellular signaling pathways and extrinsic macrophage interactions with other cells within diseased gastrointestinal tissues are still incompletely understood. Key questions include to what degree do hyperinflammatory processes and immunodeficiency intersect to drive human IBD and other inflammatory diseases, and, what is the role of macrophage heterogeneity in IBD onset and progression? Addressing such questions may inform the rational development of next generation treatments for IBD that target macrophage function.

Lack of efficacy is a source of clinical trial failure. Furthermore, mechanistic understanding of the role of drug targets in human disease is a key indicator of therapeutic success (5). Multiple drug targets, successful in mouse IBD models, have subsequently failed in clinical IBD trials (6). We therefore see future opportunities for the use of data derived from human cells and tissue, including biopsy data from normal and diseased intestinal tissues, to potentially increase the reliability and relevance of mathematical models for human IBD pathophysiology (7, 8).

The development of high-throughput experimental methods has made it possible to obtain increasingly rich data from relevant human cells and tissues. Integration of genomics, transcriptomics, proteomics, and immunohistochemistry datasets derived from macrophages and other cells requires the use of bioinformatics tools and machine learning, to organize and analyse these integrated datasets. The ever-growing availability of large-scale quantitative and structured human datasets provides a unique opportunity to rationally and systematically test hypotheses via calibrated models that may provide deeper mechanistic insights into IBD pathophysiology. In this perspective, the term “modeling” is used to describe the use of mechanistic mathematical models to conduct *in-silico* experiments, focusing on exploring macrophage roles in inflammation and tissue homeostasis. Observed discrepancies between a mathematical model and experimental data can generate biological insight by challenging assumptions on which the model is based, such as the assumption of a perfectly mixed population by Zhou et al. (discussed in Section Modeling macrophage behavior in the context of tissue microenvironments). However, the fact that a model matches a certain dataset need not generate biological insight on its own (9). We therefore propose an iterative approach of wet-lab and *in-silico* experimentation.

## Application of Mathematical Models to Inflammatory Macrophage Biology

Mathematical models have been utilized to analyse the role of macrophages in inflammatory processes and better understand macrophage intracellular signaling pathways. Relevant models were identified via PubMed and Web-of-Science searches (executed 1st January 2018) containing the words “computational” or “mathematical,” and “macrophage” or “monocyte” in their abstract and published within the last 10 years. These searches identified 605 and 736 references via PubMed and Web-of-Science. As summarized in Supplementary Table 1, sixty-one models were identified from these references by selecting mechanistic models of macrophage function in inflammation while excluding those focused on: (i) interactions between tumors and the immune system (10), (ii) macrophages in tissue repair and replacement; and (iii) the role of macrophages in debris engulfment. Although not the focus of this perspective, tissue repair, and macrophage debris engulfment are important functions in the context of the gut tissue microenvironment, with modeling conducted by Weavers et al. (11), Martin et al. (12), and Ford et al. (13) and reviewed by Dunster (14). For just over half the selected models (*n* = 31), mathematical modeling was complemented by wet-lab experimentation. The vast majority (*n* = 28/31) of associated experimental systems consisted of mouse models, murine or other animal/human immortalized cell lines. However, animal models and cell lines do not recapitulate all features of human disease pathophysiology and response to drug exposure (47). As cellular pathways are both type and species specific, we see future opportunities to develop models parametrized solely by data derived from human cells and tissue.

Note that the models listed in the table are all dynamic, describing time-dependent changes in macrophage cell numbers and/or cytokine concentrations and knowledge-driven, i.e., model development was guided and informed by relevant biology. Data-driven modeling is a more recent approach, driven by advances in computational power and the availability of large and complex data sets, including, whole genome sequencing (WGS), single cell imaging and transcriptomics derived data. As this perspective focuses on mechanistic models, no data-driven models were included in Supplementary Table 1. Machine learning techniques have been utilized to infer possible gene interaction networks from gene expression data alone, without leveraging relevant prior biological knowledge. However, gene network inference is challenging and its accuracy is low (15). Nonetheless, in the longer-term, as more complete datasets become available, these approaches may inform automated mathematical model development workflows. Examples of the many algorithms used to infer gene interaction networks from expression data [see for a comparison of methods (16)], include CLR (Context Likelihood of Relatedness) (17), ARACNe (Algorithm for the Reconstruction of Accurate Cellular Networks) (18) and GENIE3 (GEne Network Inference with Ensemble of trees) (19). ARACNe has been used to identify key genes involved in macrophage activation from a human macrophage gene expression data set generated under varying stimulatory conditions (20).

These methods are purely data-driven and produce static gene networks. However, cellular interactions are dynamic and, in part, driven by, dynamic protein interactions (e.g., signaling pathways). Furthermore, intracellular protein concentrations and related functional activity levels do not necessarily correlate with corresponding gene transcription levels (21). Accordingly, we anticipate that as gene interaction knowledge becomes richer and integrated with other data types such as proteomic data, future data-driven models will be increasingly dynamic in nature and more deeply integrated with mechanistic modeling approaches. Examples of data driven modeling techniques used in a variety of other cell types to construct dynamic gene networks from gene expression data include not only differential equation models, but also Boolean and dynamic Bayesian models [reviewed by Hecker et al. (22)]. A key challenge for data-driven modeling is integration with existing knowledge of pathway interactions, and more generally, known biological mechanisms. Of note, emerging algorithms that integrate prior knowledge of gene interactions typically outperform algorithms solely using gene expression data (15). Advances in machine learning and data driven tools, together with richer datasets, will improve our ability to identify the critical biological determinants (e.g., key cell types, interactions, proteins, and associated pathways and networks) mediating the observable behavior of human tissues and organs (e.g., human intestine) and thereby inform the development of dynamic mechanistic *in silico* models.

## Modeling Macrophage Behaviour in the Context of Tissue Microenvironments

**Figure 1:**
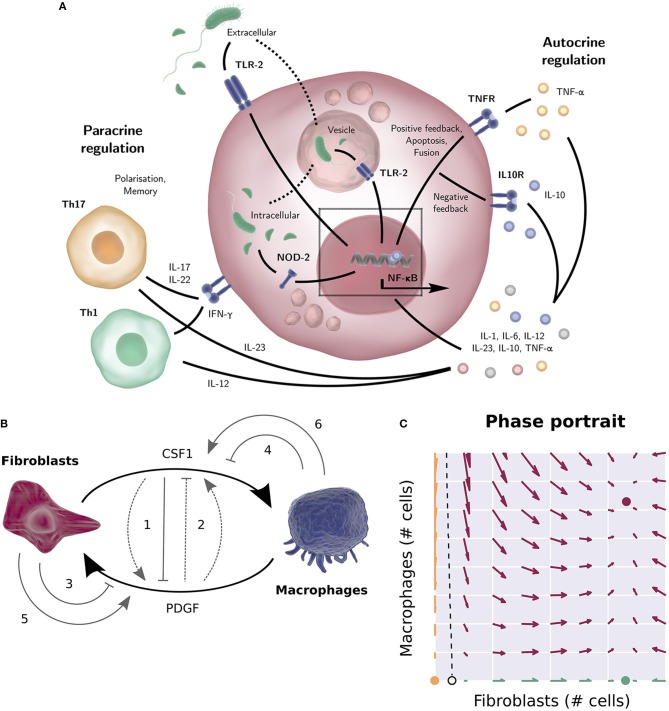
**(A)** TLR-2 can sense the bacterial product LPS both outside the cell and in vesicles, after engulfment of the bacterium. NOD-2 can sense the bacterial product MDP that is exported from vesicles (23). The NF-κB activation in response to TLR-2 or NOD-2 signaling results in the production of cytokines such as pro-inflammatory cytokine TNF-α, IL-6, or IL-8 (with positive feedback loops) or anti-inflammatory cytokine IL-10 (a negative feedback loop to downregulate inflammation). Apart from the autocrine regulation, many cytokines stimulate other cell types (IL-12 for instance drives naïve T-cells toward a Th1 phenotype, while IL-23 promotes Th17 differentiation etc.). Activated T cells in turn produce macrophage response shaping mediators themselves, such as IFN-γ, IL-17, and IL-22. **(B)** Wiring diagram of the macrophage-fibroblast growth factor model by Zhou (24) and Adler (25). Fibroblasts produce both macrophage growth factor (CSF1) and fibroblast growth factors (PDGFD, HBEGF), while macrophages produce a fibroblast growth factor (PDGFB), mediating cross talk between macrophages and stroma. The dimensionless model derived from this diagram consists of two ODEs describing the population sizes of the macrophages and fibroblasts and two algebraic equations describing the concentration of the two growth factors. Different wiring possibilities were explored (gray arrows), i.e., the addition of positive or negative feedback of one growth factor on the production rate of the other (1, 2), removal of a growth factor through receptor mediated endocytosis (3, 4), or autocrine growth factor production (5, 6). Of the 144 possibilities considered, only 48 networks allowed for a stable steady state for a wide range of parameters, corresponding to a stable number of macrophages and fibroblasts. The final experimentally tested circuit is depicted by the solid arrows. **(C)** Phase portrait of the macrophage and fibroblast cell population numbers of the model by Zhou (24) and Adler (25). Given initial cell numbers, the system will end up in one of the three stable steady states. All initial values at the left-hand side of the separatrix (dashed line) will converge to the trivial steady state (yellow, no fibroblasts or macrophages). At the right-hand side of the separatrix, the system will converge to the positive steady state if the initial system contains macrophages (red, positive numbers of fibroblasts and macrophages), and converge to the “fibroblast only” steady state (green, only fibroblasts) otherwise. Several figure components taken from the “Library of Science & Medical Illustrations” by SomerSault1824 were used in panel **(A,B)** (http://www.somersault1824.com/science-illustrations/). panel **(B,C)** are based on Zhou et al. (24), Figures 3A, 4E, 5B.

The dynamic crosstalk between macrophages and their microenvironment is key to understanding the role of macrophages in normal, healthy, and diseased, IBD gastrointestinal tissues. Their behavior depends on both their origin (tissue resident vs. monocyte derived inflammatory macrophages) and the stimuli they have previously encountered. Activated monocyte-derived macrophages have historically been identified as two mutually exclusive groups: pro-inflammatory, classically activated, M1 and anti-inflammatory, alternatively activated, M2 macrophages. Differentiation into one of these two subtypes was assumed to be driven by the different stimuli the macrophage receives within their resident tissue. Furthermore, macrophage cytokine and growth factor production modulate their microenvironment, within the intestinal lamina propria (Figure 1A). Although the binary M1/M2 framework provides a useful distinction between inflammatory and non-inflammatory (tissue repair) macrophage populations, tissue macrophages are extremely heterogeneous, existing in an essential continuum of functional states, depending on the various stimuli they have received and integrated over time (26).

Mesenchymal derived fibroblasts support the integrity of intestinal and other mucosal barriers via synthesis of extracellular matrix and growth factors required for both barrier repair and macrophage homeostasis. Recently, Ruslan Medzhitov and colleagues utilized a combination of experimentation and modeling based on an *in-vitro* system of bone-marrow derived macrophages and primary mouse embryonic fibroblasts to dissect feedback signaling loops between macrophages and stromal fibroblasts (24, 25). In this system the macrophages and fibroblasts were plated together in culture medium without addition of growth factors and cell numbers determined by flow cytometry. The mathematical model describes how simple macrophage-fibroblast interactions can reach stable cell populations. This is an illustration of how modeling can provide a useful framework for qualitative understanding of the dynamics between different cells. The model also proved useful on a quantitative level; cell-density had to be taken into consideration to match experimentally observed cell numbers predicted by the model. This in turn led to experimentally tested findings that close macrophage-fibroblast contact is essential for growth factor exchange.

Specifically, the experimentally confirmed findings were (1) fibroblasts in the system produce both macrophage and fibroblast growth factors, while the macrophages only produce a fibroblast growth factor; (2) the growth rate of the fibroblasts, but not the macrophages, is limited by their carrying capacity, which was found to be dependent on available space. Based on these two findings, a mathematical model was constructed describing macrophage and fibroblast cell counts and growth factor concentrations over time. Different wiring possibilities for the model network were explored mathematically. Of the 144 possibilities considered, only 48 networks allowed for a stable steady state across a wide range of parameters, corresponding to a stable number of macrophages and fibroblasts. It was found that all 48 networks that allowed for such a stable steady state included a negative regulatory loop on the macrophage growth factor. This is a necessary condition for stability, as a cell population that is not limited by its carrying capacity will keep expanding indefinitely if its growth factor is not regulated. Experimental studies subsequently showed that macrophage growth factor is negatively regulated by receptor internalization. Furthermore, it was found that fibroblast growth factor is also negatively regulated, both by receptor internalization and by the macrophage growth factor, however the model indicates that this regulation of fibroblast growth factor does not significantly alter system dynamics (Figure 1B). The mathematical model based on the final circuit generated in this way predicts that apart from the stable steady state, there also exists a state with only fibroblasts, sustaining themselves, and a state without macrophages and fibroblasts. Depending on the initial absolute cell numbers, the system will converge to one of these states (Figure 1C), which was experimentally tested by quantifying cell numbers over time using a combination of flow cytometry and fluorescent imaging. Finally, it was found that the initial cell numbers required to converge to the steady state of coexisting cell populations was larger than the model predicted. This was explained by density-dependent effects; the model assumes a perfectly mixed population, but cell-dependent contact decreases when cell numbers decrease. Thus, the discrepancy of the model predictions with the experimental results suggested that cell-cell contact is essential for growth factor dynamics and the regulation of tissue homeostasis.

## Modeling Macrophage Intracellular Signalling

**Figure 2:**
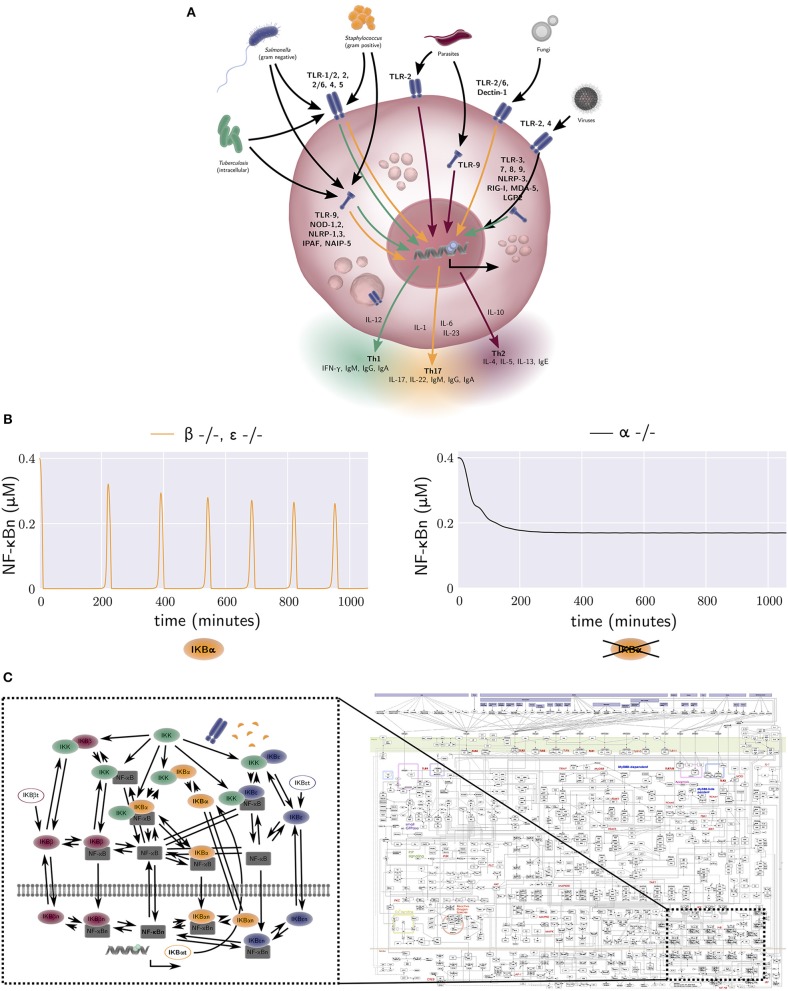
**(A)** The activation of macrophage signaling pathways by various pathogens. Macrophage output in the form of cytokine production is amongst others dependent on the type of pathogen and the receptor location. Green, yellow, and red arrows correspond to a Th1, Th17, and Th2 polarizing response, respectively. Macrophage responses exist in a continuum. **(B)** The free nuclear NF-κB concentration against time generated by the equations of the model by Alexander Hoffmann et al. (31). The model provides an explanation for the oscillatory dynamics of the nuclear NF-κB concentration that are observed in wild-type mice, but not in mice that lack an active form of IκBα. Each NF-κB inhibitor can bind to a NF-κB molecule, forming an NF-κB-inhibitor complex. When IκB kinase (IKK) also binds to this NF-κB-inhibitor complex, the inhibitor degrades, and the free NF-κB can travel to the nucleus and bind DNA. This results in the synthesis of various proteins, one of which is IκBα. The production rate of the NF-κB inhibitor IκBα is thus dependent on the concentration of free NF-κB. The negative NF-κB–IκBα feedback loop generates oscillations in the concentration of NF-κB. In contrast, the other two NF-κB inhibitors, IκBβ and IκBε, are produced at a constant rate, independent of the amount of free NF-κB. Therefore, they have a damping effect on the oscillations generated by the IκBα negative feedback loop. A model without IκBβ or IκBε, but with IκBα therefore produces oscillations (left, yellow), while a model without IκBα, but with IκBβ and IκBε does not (right, black). **(C)** Left: the wiring network from the NF-κB model by Alexander Hoffmann et al. (31). The model derived from this network consists of 26 ODEs, one for every node in the network. The interactions between nodes, denoted by arrows in the network, are included in the terms of these 26 ODEs. Right: a map of all protein interactions thought to be involved in mammal macrophage TLR signalling pathways, with the relationship of Hoffmann's NF-κB signaling model also illustrated. The map was constructed by Kanae Oda and Hiroaki Kitano (32). Several figure components taken from the “Library of Science & Medical Illustrations” by SomerSault1824 were used in **(A–C)** (http://www.somersault1824.com/science-illustrations/). Panel **(C)** is based on Oda and Kitano (32), Figure 1.

Macrophages sense and respond to their localized tissue microenvironments and in this role must integrate different external stimuli and respond appropriately. Multiple macrophage receptor systems detect specific changes in local tissue microenvironments including the presence of pathogens [Toll-like receptors and NOD-like receptors (27, 28)], cell damage [RAGE and Toll-like receptors via alarmins (29)], cytokines (cytokine receptors that detect growth factors including M-CSF, interleukins such as IL-1,6,10, tumor-necrosis factor-α, and chemokines), and neurotransmitters (30). The resulting macrophage responses may result in the production of activating and inhibitory cytokines, orchestrating the timing of pathogen specific innate and adaptive immune responses and associated intra- and extra-cellular microbial clearance (23) (Figure 2A). To better understand macrophage sensing and response behaviors, intracellular signaling network models have been constructed and used to generate experimentally testable predictions about the effect of blocking individual proteins including TLR3 (33), TLR4, TNF, IFN-β, and IL-10 (34), TLR3, TLR7, Type-1-IFNs, and IL-10 (35), TLR, JAK/STAT, and ITAM (36), and TLR, JAK/STAT and nitric oxide (37) on intracellular signaling dynamics (38). Many models were based on experimental mouse models or immortalized cell lines. Thus, the species and lineage specificity of these networks and the interacting cell types needs to be critically analyzed to understand their relevance to human IBD pathophysiology (39).

A key integrator of different macrophage signaling pathways is the NF-κB pathway, which regulates nuclear localization of NF-κB transcriptional regulators controlling expression of hundreds of genes involved in inflammation (40). One of the seminal mathematical descriptions of NF-κB signaling was developed by Hoffmann et al. This model provided a quantitative description of three NF-κB inhibitor isoforms, IκBα, IκBβ, and IκBε (31). It was one of the first studies to use an iterative approach of modeling (*in-silico* experimentation) and wet lab experimentation to better understand intracellular signaling mechanisms. The model was calibrated with data obtained from an experimental mouse model with only one active NF-κB inhibitor isoform and provides a mechanism-based explanation for the oscillatory dynamics of nuclear NF-κB concentration observed in wild-type mice, but not in mice that lack an active form of IκBα (Figure 2B). Many more mathematical models of NF-κB dependent processes were subsequently constructed, including models of TNF-α receptor signaling (41), TNF-α secretion (42), TLR4 receptor signaling, and the addition of extrinsic noise to the synthesis rate of TLR4, the activation rates of TRIF and MyD88 and the endosomal maturation rate, to incorporate cell-to-cell variability (43) [see (44) for a review of earlier models].

The above modeling frameworks (31) were developed by converting a signaling, protein interaction network diagram into a system of ODEs to quantitatively represent key reactions of the network driving dynamic changes in the concentrations of corresponding key proteins. In general, mass action, Michaelis-Menten, or Hill equation kinetics were used to derive reaction equations (45).

Static maps of all protein interactions believed to be involved in mammalian macrophage TLR signaling pathways have previously been generated [Figure 2C (Right), reproduced from Oda and Kitano (32)], with the relationship of Hoffmann's NF-κB signaling model [Figure 2C (Left)] also illustrated. The model derived from this latter network consists of 26-ODEs, one for every network node. The interactions between nodes, denoted by arrows in the network, are included in the terms for these ODEs. Advances in computational power, high-throughput data generation, data driven model parameterization and machine learning techniques will empower larger scale modeling of signaling pathways and their integration with extracellular signals. For example, high-dimensional quantitative analysis of macrophage signaling pathways in human tissue biopsies from diseased and non-diseased regions of the intestine may be used to inform model structure(s) and parameterization. There are however remaining challenges including parameter identifiability. These challenges stem from the fact that current high-throughput datasets tend to have poor temporal and spatial resolution, whereas biological systems including human intestinal tissue are often spatially heterogeneous, and relevant pathophysiological processes occur across a broad range of time scales. Nonetheless, such approaches are becoming feasible, and may allow explicit *in silico* identification of key IBD mediators and processes, driving subsequent wet-lab experimental exploration, testing, and verification.

## Future Directions

Despite increasingly rich datasets on human inflammatory processes, macrophage function is still not well understood. Open questions include: (1) how do macrophage hyperinflammatory processes and immunodeficiency intersect to produce human IBD; (2) what are the functional consequences of genetic variant burden across the multiple human polymorphisms associated with inflammatory diseases and that intersect with macrophage signaling pathways; (3) what factors and cellular processes drive granuloma formation in Crohn's disease and other granulomatous disorders; (4) what is the relationship between peripheral blood monocytes and tissue resident macrophages; (5) what is the role of macrophage heterogeneity in IBD disease dynamics; (6) what is the role of long lived tissue-resident macrophages, monocyte derived macrophages, dendritic cells, neutrophils, and non-professional APCs during active IBD inflammation and remission? Mathematical models can help answer these questions at the level of experimental design, data analysis, and interpretation.

Models can be developed to predict the effects of perturbing specific protein networks, from single cell to localized tissue pathology, through to effects on higher-level physiology. Additionally, they can identify the relative importance of bacterial handling and cytokine production in tissue pathology. Proposed mechanisms can be discarded based on simulations, and new mechanisms proposed and experimentally tested (24). Many challenges remain in both the proposed application of human datasets including tissue biopsies from healthy donors and IBD patients, and the combination of modeling with high-throughput data. Parameter identifiability is challenging due to high variability and poor spatial and temporal resolution of available human datasets. Another key challenge is data integration across different spatial and temporal scales, and, in an informative way, while selecting optimal model scope and granularity for the specific scientific questions under investigation. Furthermore, within this context one should note that the hypotheses on which mathematical models are based can only be falsified, but never proven. Therefore, mathematical modeling should be seen as an investigative tool that can be used to challenge assumptions and identify key uncertainties (46). For example, models based on different mechanisms might equally well describe an observed phenomenon and discrepancies between two such models can inform experiments to distinguish between the two alternatives (9).

## Conclusions

There is a growing body of work focused on the mathematical modeling of macrophage function, e.g., modeling intracellular signaling pathways and the dynamic cross talk between these cells and other cell types such as fibroblasts. However, to date many modeling efforts have been disconnected from wet-lab experimentation or guided by experimental work on mouse models and isolated murine and human cell lines. These experimental systems do not recapitulate important features of human gastrointestinal pathophysiology, and, therefore, are limited in the extent to which they can inform mechanistic understanding of the role of macrophages in human IBD pathophysiology. Consequently, there are many open questions about the role of macrophages in human IBD. Thus, we envision a future were mechanistic mathematical models will be based on features relevant to human disease and parametrized by richer human data sets, including high-throughput assessments of biopsy tissues taken from IBD patients with increasing spatial and temporal resolution. Furthermore, we envisage deeper integration of mechanistic modeling with experimental design whereby models are used to both inform experimental medicine study designs and provide a knowledge constrained framework for the quantitative analysis and interpretation of the resulting clinical data.

## Author Contributions

All authors listed have made a substantial, direct and intellectual contribution to the work, and approved it for publication.

### Conflict of Interest Statement

JW is an employee and shareholder in Hoffmann-La Roche AG. F Hoffman La Roche, AG, have contributed to the costs of doctoral students under the supervision of EG. The remaining authors declare that the research was conducted in the absence of any commercial or financial relationships that could be construed as a potential conflict of interest.
