# Supplementary material for: Combining Mathematical Models With Experimentation to Drive Novel Mechanistic Insights Into Macrophage Function
Source: Front Immunol. 2019 Jun 6;10:1283. doi: 10.3389/fimmu.2019.01283 (PMC6563075; doi:10.3389/fimmu.2019.01283)
Supplement: Supplementary file 1 [file Data_Sheet_1.PDF]

Supplementary Material

Combining Mathematical Models with Experimentation to Drive Novel Mechanistic Insights into Macrophage Function

Joanneke E. Jansen *, Eamonn A. Gaffney, Jonathan Wagg, Mark C. Coles

*** Correspondence:** Joanneke E. Jansen**:** jansen@maths.ox.ac.uk

Supplementary Table. PubMed and Web of Science literature searches executed on 1 January 2018 for studies containing the words ‘computational’ or ‘mathematical’, and ‘macrophage’ or ‘monocyte’ in their abstract and published within the last ten years were completed. These searches identified 605 and 736 references from PubMed and Web of Science, respectively. We excluded models that focused on: (i) macrophages in tissue repair and replacement; (ii) macrophage engulfment of debris (these areas have recently been reviewed in (Dunster 2016)); (iii) interactions between tumours and the immune system (as reviewed in (Eftimie, Bramson, and Earn 2011)).

An overview of sixty-one representative models selected from the identified references was tabulated. Most tabulated models are based on coupled ordinary differential equations (ODEs) where changes in the concentration of different species of interest, including cell types, growth factors and cytokines are tracked over time. These models are deterministic (their future state is fully determined by their current state) and not dependent on space, although a finite number of spatial compartments, each with possibly different concentrations of the same types of species, can be generated. Such an approach was used in a multi-scale model of a *Helicobacter pylori* infection, with compartments for the lumen, epithelium, lamina propria and gastric lymph node, each with separate concentrations for the various cell types of interest (Leber et al. 2016). A second type of model were Agent Based Models (ABMs) where the interactions of rule-based agents lead to stochastic outcomes. This approach was used to study the role of macrophages in *Mycobacterium tuberculosis* (TB) and visceral *Leishmania major* infections (e.g. Marino et al. 2015; Moyo et al. 2018). In ABMs cells are described as individual agents leading to emergent behaviors in both time and space, as illustrated by Marino S et al., where the agents have included T cells and macrophages (Marino et al. 2015). Both approaches have been utilized to study intracellular signaling dynamics and macrophages in relation to their environment.

Study	Key subject	Model type	Scale	Number of equations	Combined with experiments on murine cells	Combined with experiments on human cells
(Salim, Sershen, and May 2016)	Pathway interaction between LPS, TNF, IFN-gamma and their effect on iNOS induction	ODE	Intracellular	114	No	No
(Day, Friedman, and Schlesinger 2009)	Macrophage repolarization rates during a *Mycobacterium tuberculosis* infection	ODE	Intercellular	11	No	No
(Siewe et al. 2016)	*Leishmania* infection model	ODE	Intercellular	14	No	No
(Hao, Schlesinger, and Friedman 2016)	Granuloma modelling during a *Mycobacterium tuberculosis* infection	PDE	Intercellular	21	No	No
(Marino et al. 2015)	Macrophage polarization during a *Mycobacterium tuberculosis* infection	Hybrid ABM	Intercellular		No	No
(Álvarez et al. 2017)	Endotoxin tolerance in human monocytes	ODE	Intercellular	6	No	Yes, peripheral blood from patients with sepsis and healthy volunteers
(Eftimie and Hamam 2017)	Macrophage repolarization rates and its role in cancer	ODE	Intercellular	5	No	No
(T. D. Smith et al. 2016)	Comparing various small network topologies of macrophage polarization	ODE	Intracellular	7	Yes, murine bone marrow derived macrophages	No
(den Breems and Eftimie 2016)	Macrophage repolarization rates and its role in cancer	ODE	Intercellular	6	No	No
(Louzoun et al. 2014)	Pancreatic cancer	ODE	Intercellular	11	No	No
(Wang et al. 2012)	Left ventricular remodeling post-myocardial infarction	ODE	Intercellular	6	Yes, monocyte and myocyte densities post myocardial	No

Study	Key subject	Model type	Scale	Number of equations	Combined with experiments on murine cells	Combined with experiments on human cells
					infection in C57 mice	
(Fu, Jiang, et al. 2012)	IFN-gamma mediated priming in human macrophages	ODE	Intracellular	36	No	No
(Fu, Glaros, et al. 2012)	Network topologies and dynamics leading to endotoxin tolerance and priming	ODE	Intracellular	3	Yes, murine bone marrow derived macrophages	No
(Nagaraja et al. 2014)	Model of acute and chronic local inflammation	ODE	Intercellular	16	No	No
(Nagaraja, Reifman, and Mitrophanov 2015)	Timing and intensity of the inflammatory response	ODE	Intercellular	16	No	No
(Lee, Adler, and Kim 2017)	Macrophage response to viral infection in normal and asthmatic conditions	ODE	Intercellular	10	No	No
(Leonard et al. 2017)	Drug-delivering macrophages and M1/M2 polarization in cancer treatment	PDE	Intercellular	5	Yes, murine bone marrow derived macrophages and breast cancer 4T1 cells	No
(Soni, Saha, and Singh 2017)	IL-6 signalling during a *Leishmania major* infection	ODE	Intracellular	42	No	No
(Mehrotra, Rao, and Chatterjee 2017)	*Mycobacterium tuberculosis* infection and mitochondrial pyruvate transporter activity	ODE	Intracellular	2	No	Yes, monocytic cell line THP-1
(Leber et al. 2016)	Role of Lanthionine Synthetase C-Like 2 during a *Helicobacter pylori* infection	ODE	Intercellular	36	Yes, stomachs of mice infected with *H. Pylori*	No

Study	Key subject	Model type	Scale	Number of equations	Combined with experiments on murine cells	Combined with experiments on human cells
(Hayes et al. 2016)	NF-κB signalling during a *Cryptococcus neoformans* infection	ODE	Intracellular	14	Yes, murine macrophages (RAW 264.7)	No
(Lalande et al. 2016)	*Mycobacterium tuberculosis* infection and its early treatment by Isoniazid in the human lung	ODE	Intercellular	21	No	No
(Oremland et al. 2016)	*Aspergillosis* infection model and the role of iron	ABM	Intercellular		Yes, murine model infected with *Aspergillosis fumigatus*	No
(Pawelek et al. 2016)	The role of macrophages in influenza virus infections	ODE	Intercellular	7	No	No
(Philipson et al. 2015)	NLRX1 signalling during a *Helicobacter pylori* infection	ODE	Intercellular	15	Yes, murine bone marrow derived macrophages and murine model of *Helicobacter pylori* infection	No
(Tanaka et al. 2015)	Model of fungal spore inhalation	ODE	Intercellular	4	Yes, murine model infected with *Aspergillosis fumigatus*	No
(Pedruzzi, Rao, and Chatterjee 2015)	Host-pathogen interaction during *Mycobacterium tuberculosis* infection	ODE	Intercellular	4	No	No
(Linderman et al. 2015)	Multiscale model of a *Mycobacterium tuberculosis* infection	Hybrid ABM	Intercellular		No	No

Study	Key subject	Model type	Scale	Number of equations	Combined with experiments on murine cells	Combined with experiments on human cells
(Cilfone, Pienaar, et al. 2015)	*Mycobacterium tuberculosis* infection and inhaled antibiotics	Hybrid ABM	Intercellular		No	No
(Cilfone, Ford, et al. 2015)	The role of IL-10 during *Mycobacterium tuberculosis*infection	Hybrid ABM	Intercellular		No	No
(Gillard et al. 2014)	Model of a *Francisella tularensis* infection	Stochastic	Intercellular		No	No
(Gog et al. 2012)	Model of a *Salmonella* infection	ODE	Intracellular	10	Yes, murine bone marrow derived macrophages and RAW 264.7 cells	No
(Fallahi-Sichani, Kirschner, and Linderman 2012)	NF-κB signalling during a *Mycobacterium tuberculosis*infection	Hybrid ABM	Intercellular		No	No
(Leander et al. 2012)	CR3/TLR2 crosstalk during a *Francisella tularensis* infection	ODE	Intercellular	9	No	No
(A. M. Smith, McCullers, and Adler 2011)	Model of a *Streptococcus pneumoniae* lung infection	ODE	Intercellular	7	Yes, murine model infected with Streptococcus pneumoniae	No
(Fallahi-Sichani, Kirschner, and Linderman 2012)	The role of TNF-alpha during *Mycobacterium tuberculosis*infection	Hybrid ABM	Intercellular		No	No
(Klotz et al. 2011)	The interaction between AvCystatin and the IL-10 signalling pathway	ODE	Intracellular	15	Yes, murine peritoneal macrophages and a murine model treated with AvCystatin	No

Study	Key subject	Model type	Scale	Number of equations	Combined with experiments on murine cells	Combined with experiments on human cells
(Marino et al. 2010)	TNF and IL-10 signalling during a *Mycobacterium tuberculosis*infection	ODE	Intercellular	32	Yes, murine model infected with *Mycobacterium tuberculosis*	No
(Ray, Flynn, and Kirschner 2009)	TNF signalling during a *Mycobacterium tuberculosis*infection	Hybrid ABM	Intercellular		No	No
(Ray et al. 2008)	TNF and IFN-gamma signalling during a *Mycobacterium tuberculosis*infection	ODE	Intracellular	10	No	No
(Mol, Patole, and Singh 2013)	Macrophage signalling during a *Leishmania* infection	ODE	Intracellular	65	No	No
(Carvalho et al. 2015)	Petri net model of a *Mycobacterium tuberculosis* infection	Petri net	Intercellular		No	No
(Hünniger et al. 2014)	Interaction between neutrophils, macrophages and *Candida albicans*	State based model	Intercellular		No	Yes, primary human blood mononuclear cells
(Figueiredo et al. 2009)	Interaction between nematode-derived cytastin and the IL-10 pathway	ODE	Intracellular	10	Yes, murine thioglycollate-elicited peritoneal macrophages	No
(Kulawik et al. 2017)	Comparison of MK2 responsiveness towards IL-1beta between hepatocytes and macrophages	ODE	Intracellular	5	Yes, murine bone marrow derived macrophages and primary mouse hepatocytes	No
(Li et al. 2017)	Model of TLR3 and TLR7 pathway crosstalk	ODE	Intracellular	112	Yes, murine bone marrow derived macrophages	No

Study	Key subject	Model type	Scale	Number of equations	Combined with experiments on murine cells	Combined with experiments on human cells
(Junkin et al. 2016)	LPS induced single cell NF-κB dynamics	ODE (including extrinsic noise)	Intracellular	48	Yes, single murine macrophages (RAW 264.7)	No
(Saul et al. 2016)	Interaction between calcium channels and oxidative burst	ODE	Intracellular	1	Yes, an acute lung infection model of mice with S. aureus	Yes, primary human monocytes
(Tomaiuolo et al. 2016)	TNF production networks and interaction with IL-10	ODE	Intracellular	78	No	No
(Anderson et al. 2015)	Cytokine signalling in neural macrophages	ODE	Cytokines	12	Yes, murine bone marrow derived macrophages	No
(Z. Cheng et al. 2015)	LPS induced single cell signaling characteristics	ODE (including extrinsic noise)	Intracellular	48	Yes, murine bone marrow derived macrophages and RAW 264.7 cells	No
(Ourthiague et al. 2015)	Specificity of the transcription factors IRF3 and ISGF3	ABM	Intercellular		Yes, murine bone marrow derived macrophages	No
(Sung et al. 2014)	LPS induced single cell NF-κB dynamics	ODE	Intracellular	14	Yes, murine macrophages (RAW 264.7)	No
(Namas et al. 2013)	Comparison of inflammation between middle-aged and young mice	ODE	Intracellular	17	Yes, murine blood samples and resident peritoneal cells	No

**Study**	**Key subject**	**Model type**	**Scale**	**Number of equations**	**Combined with experiments on murine cells**	**Combined with experiments on human cells**
(C. S. Cheng et al. 2011)	Cross-regulation between NF-κB and IRF	ODE	Intracellular	1	Yes, murine bone marrow derived macrophages	No
(Helmy et al. 2009)	Macrophage TLR3 interactions	ODE	Intracellular	34	No	No
(Selvarajoo et al. 2008)	TLR4 signalling and signaling flux redistribution	ODE	Intracellular	30	Yes, murine spleen derived macrophages	No
(Schulz et al. 2017)	NF-κB signalling during a *Legionella pneumophila* infection	ODE	Intracellular	10	No	Yes, alveolar type II cell line A549 and monocytic cell line THP-1
(Chatterjee et al. 2016)	TLR4 signalling and the canonical and non-canonical NF-κB signalling pathways	ODE	Intracellular	36	Yes, murine bone marrow derived macrophages	No
(C. S. Cheng et al. 2017)	Transcription factor interactions	ODE	Intracellular	17	Yes, murine bone marrow derived macrophages and murine fibroblasts (MEFs)	No
(Maiti et al. 2014)	Model of TLR4, TNFR and IL10R pathway crosstalk	ODE	Intracellular	29	No	No

References

Álvarez, Enrique, Víctor Toledano, Fernando Morilla, Enrique Hernández-Jiménez, Carolina Cubillos-Zapata, Aníbal Varela-Serrano, José Casas-Martín, et al. 2017. “A System Dynamics Model to Predict the Human Monocyte Response to Endotoxins.” *Frontiers in*

Immunology 8 (AUG). https://doi.org/10.3389/fimmu.2017.00915.

Anderson, Warren D., Hirenkumar K. Makadia, Andrew D. Greenhalgh, James S. Schwaber, Samuel David, and Rajanikanth Vadigepalli. 2015. "Computational Modeling of Cytokine Signaling in Microglia." *Molecular BioSystems* 11 (12): 3332–46. https://doi.org/10.1039/c5mb00488h.

Breems, Nicoline Y. den, and Raluca Eftimie. 2016. "The Re-Polarisation of M2 and M1 Macrophages and Its Role on Cancer Outcomes." *Journal of Theoretical Biology* 390: 23–39. https://doi.org/10.1016/j.jtbi.2015.10.034.

Carvalho, Rafael, Jeroen van den Heuvel, Jetty Kleijn, and Fons Verbeek. 2015. "Coupling of Petri Net Models of the Mycobacterial Infection Process and Innate Immune Response." *Computation* 3 (2): 150–76. https://doi.org/10.3390/computation3020150.

Chatterjee, Budhaditya, Balaji Banoth, Tapas Mukherjee, Nandaraj Taye, Bharath Vijayaragavan, Samit Chattopadhyay, James Gomes, and Soumen Basak. 2016. "Late-Phase Synthesis of IκBα Insulates the TLR4-Activated Canonical NF-KB Pathway from Noncanonical NF-KB Signaling in Macrophages." *Science Signaling* 9 (457). https://doi.org/10.1126/scisignal.aaf1129.

Cheng, Christine S., Marcelo S. Behar, Gajendra W. Suryawanshi, Kristyn E. Feldman, Roberto Spreafico, and Alexander Hoffmann. 2017. "Iterative Modeling Reveals Evidence of Sequential Transcriptional Control Mechanisms." *Cell Systems* 4 (3): 330–343.e5. https://doi.org/10.1016/j.cels.2017.01.012.

Cheng, Christine S., Kristyn E. Feldman, James Lee, Shilpi Verma, De-Bin Bin Huang, Kim Huynh, Mikyoung Chang, et al. 2011. "The Specificity of Innate Immune Responses Is Enforced by Repression of Interferon Response Elements by NF-KB P50." *Science Signaling* 4 (161). https://doi.org/10.1126/scisignal.2001501.

Cheng, Zhang, Brooks Taylor, Diana R Ourthiague, and Alexander Hoffmann. 2015. "Distinct Single-Cell Signaling Characteristics Are Conferred by the MyD88 and TRIF Pathways during TLR4 Activation." *Science Signaling* 8 (385): 1–13. https://doi.org/10.1126/scisignal.aaa5208.

Cilfone, Nicholas A., Christopher B. Ford, Simeone Marino, Joshua T. Mattila, Hannah P. Gideon, JoAnne L. Flynn, Denise E. Kirschner, and Jennifer J. Linderman. 2015. "Computational Modeling Predicts IL-10 Control of Lesion Sterilization by Balancing Early Host Immunity–Mediated Antimicrobial Responses with Caseation during Mycobacterium Tuberculosis Infection." *The Journal of Immunology* 194 (2): 664–77. https://doi.org/10.4049/jimmunol.1400734.

Cilfone, Nicholas A., Elsje Pienaar, Greg M. Thurber, Denise E. Kirschner, and Jennifer J. Linderman. 2015. "Systems Pharmacology Approach toward the Design of Inhaled Formulations of Rifampicin and Isoniazid for Treatment of Tuberculosis." *CPT: Pharmacometrics and Systems Pharmacology* 4 (3): 193–203. https://doi.org/10.1002/psp4.22.

Day, Judy, Avner Friedman, and L.S. Larry S Schlesinger. 2009. "Modeling the Immune Rheostat of Macrophages in the Lung in Response to Infection." *Proceedings of the National Academy of Sciences of the United States of America* 106 (27): 11246–51. https://doi.org/10.1073/pnas.0904846106.

Dunster, Joanne L. 2016. "The Macrophage and Its Role in Inflammation and Tissue Repair: Mathematical and Systems Biology Approaches." *Wiley Interdisciplinary Reviews: Systems Biology and Medicine* 8 (1): 87–99. https://doi.org/10.1002/wsbm.1320.

Eftimie, Raluca, Jonathan L. Bramson, and David J.D. Earn. 2011. "Interactions between the Immune System and Cancer: A Brief Review of Non-Spatial Mathematical Models." *Bulletin of Mathematical Biology*. Springer-Verlag. https://doi.org/10.1007/s11538-010-9526-3.

Eftimie, Raluca, and Haneen Hamam. 2017. "Modelling and Investigation of the CD4+T Cells – Macrophages Paradox in Melanoma Immunotherapies." *Journal of Theoretical Biology* 420: 82–104. https://doi.org/10.1016/j.jtbi.2017.02.022.

Fallahi-Sichani, Mohammad, Denise E. Kirschner, and Jennifer J. Linderman. 2012. "NF-KB Signaling Dynamics Play a Key Role in Infection Control in Tuberculosis." *Frontiers in Physiology* 3 JUN. https://doi.org/10.3389/fphys.2012.00170.

Figueiredo, Ana Sofia A.S. S, Thomas Höfer, Christian Klotz, Christine Sers, Susanne Hartmann, Richard Lucius, and Peter Hammerstein. 2009. "Modelling and Simulating Interleukin-10 Production and Regulation by Macrophages after Stimulation with an Immunomodulator of Parasitic Nematodes." *FEBS Journal* 276 (13): 3454–69. https://doi.org/10.1111/j.1742-4658.2009.07068.x.

Fu, Yan, Trevor Glaros, Meng Zhu, Ping Wang, Zhanghan Wu, John Tyson, Liwu Li, and Jianhua Xing. 2012. "Network Topologies and Dynamics Leading to Endotoxin Tolerance and Priming in Innate Immune Cells." *PLoS Computational Biology* 8 (5). https://doi.org/10.1371/journal.pcbi.1002526.

Fu, Yan, Xiaoshan Jiang, Hang Zhang, and Jianhua Xing. 2012. "A Strategy to Study Pathway Cross-Talks of Cells under Repetitive Exposure to Stimuli." *BMC Systems Biology* 6 (SUPPL3). https://doi.org/10.1186/1752-0509-6-S3-S6.

Gillard, Joseph, Thomas R. Laws, Grant Lythe, and Carmen Molina-París. 2014. "Modelling Early Events in Francisella Tularensis Pathogenesis." *Frontiers in Cellular and Infection Microbiology* 4 (NOV). https://doi.org/10.3389/fcimb.2014.00169.

Gog, Julia R, Alicia Murcia, Natan Osterman, Olivier Restif, Trevelyan J. McKinley, Mark Sheppard, Sarra. Achouri, et al. 2012. "Dynamics of Salmonella Infection of Macrophages at the Single Cell Level." *Journal of the Royal Society Interface* 9 (75): 2696–2707. https://doi.org/10.1098/rsif.2012.0163.

Hao, Wenrui, Larry S. Schlesinger, and Avner Friedman. 2016. "Modeling Granulomas in Response to Infection in the Lung." *PLoS ONE*

11 (3). https://doi.org/10.1371/journal.pone.0148738.

Hayes, James B, Linda M Sircy, Lauren E Heusinkveld, Wandi Ding, Rachel N Leander, Erin E McClelland, and David E Nelson. 2016. “Modulation of Macrophage Inflammatory Nuclear Factor KB (NF-KB) Signaling by Intracellular Cryptococcus Neoformans.” *Journal of Biological Chemistry* 291 (30): 15614–27. https://doi.org/10.1074/jbc.M116.738187.

Helmy, Mohamed, Jin Gohda, Jun-ichiro J.-I. Inoue, Masaru Tomita, Masa Tsuchiya, and Kumar Selvarajoo. 2009. “Predicting Novel Features of Toll-like Receptor 3 Signaling in Macrophages.” *PLoS ONE* 4 (3): e4661. https://doi.org/10.1371/journal.pone.0004661.

Hünniger, Kerstin, Teresa Lehnert, Kristin Bieber, Ronny Martin, Marc Thilo Figge, and Oliver Kurzai. 2014. “A Virtual Infection Model Quantifies Innate Effector Mechanisms and Candida Albicans Immune Escape in Human Blood.” *PLoS Computational Biology* 10 (2). https://doi.org/10.1371/journal.pcbi.1003479.

Junkin, Michael, Alicia J. Kaestli, Zhang Cheng, Christian Jordi, Cem Albayrak, Alexander Hoffmann, and Sava Tay. 2016. “High-Content Quantification of Single-Cell Immune Dynamics.” *Cell Reports* 15 (2): 411–22. https://doi.org/10.1016/j.celrep.2016.03.033.

Klotz, Christian, Thomas Ziegler, Ana Sofia A.S. S Figueiredo, Sebastian Rausch, Matthew R. M.R. Hepworth, Nadja Obsivac, Christine Sers, et al. 2011. “A Helminth Immunomodulator Exploits Host Signaling Events to Regulate Cytokine Production in Macrophages.” *PLoS Pathogens* 7 (1). https://doi.org/10.1371/journal.ppat.1001248.

Kulawik, Andreas, Raphael Engesser, Christian Ehlting, Andreas Raue, Ute Albrecht, Bettina Hahn, Wolf-Dieter Lehmann, et al. 2017. “IL-1-Induced and P38MAPK-Dependent Activation of the Mitogen-Activated Protein Kinase-Activated Protein Kinase 2 (MK2) in Hepatocytes: Signal Transduction with Robust and Concentration-Independent Signal Amplification.” *Journal of Biological Chemistry* 292 (15): 6291–6302. https://doi.org/10.1074/jbc.M117.775023.

Lalande, Laure, Laurent Bourguignon, Pascal Maire, and Sylvain Goutelle. 2016. “Mathematical Modeling and Systems Pharmacology of Tuberculosis: Isoniazid as a Case Study.” *Journal of Theoretical Biology* 399: 43–52. https://doi.org/10.1016/j.jtbi.2016.03.038.

Leander, Rachel, Shipan Dai, L.S. Larry S. Schlesinger, and Avner Friedman. 2012. “A Mathematical Model of CR3/TLR2 Crosstalk in the Context of Francisella Tularensis Infection.” Edited by Rob J. De Boer. *PLoS Computational Biology* 8 (11): e1002757. https://doi.org/10.1371/journal.pcbi.1002757.

Leber, Andrew, Josep Bassaganya-Riera, Nuria Tubau-Juni, Victoria Zoccoli-Rodriguez, Monica Viladomiu, Vida Abedi, Pinyi Lu, and Raquel Hontecillas. 2016. “Modeling the Role of Lanthionine Synthetase C-Like 2 (LANCL2) in the Modulation of Immune Responses to Helicobacter Pylori Infection.” *PLoS ONE* 11 (12). https://doi.org/10.1371/journal.pone.0167440.

Lee, Junehyuk, Frederick R. Adler, and Peter S. Kim. 2017. “A Mathematical Model for the Macrophage Response to Respiratory Viral Infection in Normal and Asthmatic Conditions.” *Bulletin of Mathematical Biology* 79 (9): 1979–98. https://doi.org/10.1007/s11538-017-0315-0.

Leonard, Fransisca, Louis T. Curtis, Matthew James Ware, Taraz Nosrat, Xuewu Liu, Kenji Yokoi, Hermann B. Frieboes, and Biana Godin. 2017. “Macrophage Polarization Contributes to the Anti-Tumoral Efficacy of Mesoporous Nanovectors Loaded with Albumin-Bound Paclitaxel.” *Frontiers in Immunology* 8 (JUN). https://doi.org/10.3389/fimmu.2017.00693.

Li, Bi-Rong, Lin-Qin Xia, Jing Liu, Yang Zhang, Min Deng, Hui-Juan Zhong, Ting-Ting Feng, Ping-Ping He, and Xin-Ping Ouyang. 2017. “MiR-758-5p Regulates Cholesterol Uptake via Targeting the CD36 3′UTR.” *Biochemical and Biophysical Research Communications* 494 (1–2): 384–89. https://doi.org/10.1016/j.bbrc.2017.09.150.

Linderman, Jennifer J., Nicholas A. Cilfone, Elsje Pienaar, Chang Gong, and Denise E. Kirschner. 2015. “A Multi-Scale Approach to Designing Therapeutics for Tuberculosis.” *Integr. Biol.* 7 (5): 591–609. https://doi.org/10.1039/C4IB00295D.

Louzoun, Yoram, Chuan Xue, Gregory B. Lesinski, and Avner Friedman. 2014. “A Mathematical Model for Pancreatic Cancer Growth and Treatments.” *Journal of Theoretical Biology* 351: 74–82. https://doi.org/10.1016/j.jtbi.2014.02.028.

Maiti, Shreya, Wei Dai, Robert Alaniz, Juergen Hahn, and Arul Jayaraman. 2014. “Mathematical Modeling of Pro- and Anti-Inflammatory Signaling in Macrophages.” *Processes* 3 (1): 1–18. https://doi.org/10.3390/pr3010001.

Marino, Simeone, Nicholas A. Cilfone, Joshua T. Mattila, Jennifer J. Linderman, Joanne L. Flynn, and Denise E. Kirschner. 2015. “Macrophage Polarization Drives Granuloma Outcome during Mycobacterium Tuberculosis Infection.” *Infection and Immunity* 83 (1): 324–38. https://doi.org/10.1128/IAI.02494-14.

Marino, Simeone, Amy Myers, JoAnne L. Flynn, and Denise E. Kirschner. 2010. “TNF and IL-10 Are Major Factors in Modulation of the Phagocytic Cell Environment in Lung and Lymph Node in Tuberculosis: A next-Generation Two-Compartmental Model.” *Journal of Theoretical Biology* 265 (4): 586–98. https://doi.org/10.1016/j.jtbi.2010.05.012.

Mehrotra, Parul, Kanury V.S. Rao, and Samrat Chatterjee. 2017. “A Mathematical Model Predicting Host Mitochondrial Pyruvate Transporter Activity to Be a Critical Regulator of Mycobacterium Tuberculosis Pathogenicity.” *BioSystems* 155: 1–9. https://doi.org/10.1016/j.biosystems.2017.02.003.

Mol, Milsee, Milind S. Patole, and Shailza Singh. 2013. “Signaling Networks in Leishmania Macrophages Deciphered through Integrated Systems Biology: A Mathematical Modeling Approach.” *Systems and Synthetic Biology* 7 (4): 185–95. https://doi.org/10.1007/s11693-

013-9111-9.

Nagaraja, Sridevi, Jaques Reifman, and Alexander Y. Mitrophanov. 2015. “Computational Identification of Mechanistic Factors That Determine the Timing and Intensity of the Inflammatory Response.” *PLoS Computational Biology* 11 (12). https://doi.org/10.1371/journal.pcbi.1004460.

Nagaraja, Sridevi, Anders Wallqvist, Jaques Reifman, and A.Y. Y Alexander Y Mitrophanov. 2014. “Computational Approach to Characterize Causative Factors and Molecular Indicators of Chronic Wound Inflammation.” *Journal of Immunology* 192 (4): 1824–34. https://doi.org/10.4049/jimmunol.1302481.

Namas, Rami A., John Bartels, Rosemary Hoffman, Derek Barclay, Timothy R. Billiar, Ruben Zamora, and Yoram Vodovotz. 2013. “Combined in Silico, in Vivo, and in Vitro Studies Shed Insights into the Acute Inflammatory Response in Middle-Aged Mice.” *PLoS ONE* 8 (7). https://doi.org/10.1371/journal.pone.0067419.

Oremland, Matthew, Kathryn R. Michels, Alexandra M. Bettina, Chris Lawrence, Borna Mehrad, and Reinhard Laubenbacher. 2016. “A Computational Model of Invasive Aspergillosis in the Lung and the Role of Iron.” *BMC Systems Biology* 10 (1). https://doi.org/10.1186/s12918-016-0275-2.

Ourthiague, Diana R., Harry Birnbaum, Niklas Ortenlof, Jesse D. Vargas, Roy Wollman, and Alexander Hoffmann. 2015. “Limited Specificity of IRF3 and ISGF3 in the Transcriptional Innate-Immune Response to Double-Stranded RNA.” *Journal of Leukocyte Biology* 98 (1): 119–28. https://doi.org/10.1189/jlb.4A1014-483RR.

Pawelek, Kasia A., Daniel Dor, Cristian Salmeron, and Andreas Handel. 2016. “Within-Host Models of High and Low Pathogenic Influenza Virus Infections: The Role of Macrophages.” *PLoS ONE* 11 (2). https://doi.org/10.1371/journal.pone.0150568.

Pedruzzi, Gabriele, Kanury V.S. Rao, and Samrat Chatterjee. 2015. “Mathematical Model of Mycobacterium-Host Interaction Describes Physiology of Persistence.” *Journal of Theoretical Biology* 376: 105–17. https://doi.org/10.1016/j.jtbi.2015.03.031.

Philipson, Casandra W., Josep Bassaganya-Riera, Monica Viladomiu, Barbara Kronsteiner, Vida Abedi, Stefan Hoops, Pawel Michalak, Lin Kang, Stephen E. Girardin, and Raquel Hontecillas. 2015. “Modeling the Regulatory Mechanisms by Which NLRX1 Modulates Innate Immune Responses to Helicobacter Pylori Infection.” *PLoS ONE* 10 (9). https://doi.org/10.1371/journal.pone.0137839.

Ray, Christian J., Joanne L. Flynn, and Denise E. Kirschner. 2009. “Synergy between Individual TNF-Dependent Functions Determines Granuloma Performance for Controlling Mycobacterium Tuberculosis Infection.” *Journal of Immunology* 182 (6): 3706–17. https://doi.org/10.4049/jimmunol.0802297.

Ray, Christian J, Jian Wang, John Chan, and Denise E. Kirschner. 2008. "The Timing of TNF and IFN-γ Signaling Affects Macrophage Activation Strategies during Mycobacterium Tuberculosis Infection." *Journal of Theoretical Biology* 252 (1): 24–38. https://doi.org/10.1016/j.jtbi.2008.01.010.

Salim, Taha, Cheryl L. Sershen, and Elebeoba E. May. 2016. "Investigating the Role of TNF-α and IFN-γ Activation on the Dynamics of INOS Gene Expression in Lps Stimulated Macrophages." *PLoS ONE* 11 (6). https://doi.org/10.1371/journal.pone.0153289.

Saul, Stephanie, Christine S. Gibhardt, Barbara Schmidt, Annette Lis, Bastian Pasieka, David Conrad, Philipp Jung, et al. 2016. "A Calcium-Redox Feedback Loop Controls Human Monocyte Immune Responses: The Role of ORAI Ca2+ Channels." *Science Signaling* 9 (418). https://doi.org/10.1126/scisignal.aaf1639.

Schulz, Christine, Xin Lai, Wilhelm Bertrams, Anna Lena A.L. Jung, Alexandra Sittka-Stark, C.E. Christina Elena Herkt, Harshavadhan Janga, et al. 2017. "THP-1-Derived Macrophages Render Lung Epithelial Cells Hypo-Responsive to Legionella Pneumophila - A Systems Biology Study." *Scientific Reports* 7 (1). https://doi.org/10.1038/s41598-017-12154-4.

Selvarajoo, Kumar, Yasunari Takada, Jin Gohda, Mohamed Helmy, Shizuo Akira, Masaru Tomita, Masa Tsuchiya, Jun Ichiro J.-I. Inoue, and Koichi Matsuo. 2008. "Signaling Flux Redistribution at Toll-like Receptor Pathway Junctions." *PLoS ONE* 3 (10). https://doi.org/10.1371/journal.pone.0003430.

Siewe, Nourridine, Abdul-Aziz Yakubu, Abhay R. Satoskar, and Avner Friedman. 2016. "Immune Response to Infection by Leishmania: A Mathematical Model." *Mathematical Biosciences* 276: 28–43. https://doi.org/10.1016/j.mbs.2016.02.015.

Smith, Amber M., Jonathan A. McCullers, and Frederick R. Adler. 2011. "Mathematical Model of a Three-Stage Innate Immune Response to a Pneumococcal Lung Infection." *Journal of Theoretical Biology* 276 (1): 106–16. https://doi.org/10.1016/j.jtbi.2011.01.052.

Smith, Tim D., Margaret J. Tse, Elizabeth L. Read, and Wendy F. Liu. 2016. "Regulation of Macrophage Polarization and Plasticity by Complex Activation Signals." *Integr. Biol.* 8 (9): 946–55. https://doi.org/10.1039/C6IB00105J.

Soni, Bhavnita, Bhaskar Saha, and Shailza Singh. 2017. "Systems Cues Governing IL6 Signaling in Leishmaniasis." *Cytokine*, 2017. https://doi.org/10.1016/j.cyto.2017.11.001.

Sung, Myong Hee, Ning Li, Qizong Lao, Rachel A. Gottschalk, Gordon L. Hager, and Iain D C Fraser. 2014. "Switching of the Relative Dominance between Feedback Mechanisms in Lipopolysaccharide-Induced NF-KB Signaling." *Science Signaling* 7 (308). https://doi.org/10.1126/scisignal.2004764.

Tanaka, Reiko J., Neville J. Boon, Katarina Vrcelj, Anita Nguyen, Carmelina Vinci, Darius Armstrong-James, and Elaine Bignell. 2015. "In Silico Modeling of Spore Inhalation Reveals Fungal Persistence Following Low Dose Exposure." *Scientific Reports* 5. https://doi.org/10.1038/srep13958.

Tomaiuolo, Maurizio, Melissa Kottke, Ronald W. Matheny, Jaques Reifman, and Alexander Y. Mitrophanov. 2016. "Computational Identification and Analysis of Signaling Subnetworks with Distinct Functional Roles in the Regulation of TNF Production." *Molecular BioSystems* 12 (3): 826–38. https://doi.org/10.1039/c5mb00456j.

Wang, Yunji, Tianyi Yang, Yonggang Ma, Ganesh V. Halade, Jianqiu Zhang, Merry L. Lindsey, and Yu-Fang F. Jin. 2012. "Mathematical Modeling and Stability Analysis of Macrophage Activation in Left Ventricular Remodeling Post-Myocardial Infarction." *BMC Genomics* 13 Suppl 6 (Suppl 6): S21. https://doi.org/10.1186/1471-2164-13-S6-S21.
